# Task Specificity of Dynamic Resistance Training and Its Transferability to Non-trained Isometric Muscle Strength: A Systematic Review with Meta-analysis

**DOI:** 10.1007/s40279-025-02225-2

**Published:** 2025-05-02

**Authors:** Atle H. Saeterbakken, Nicolay Stien, Gøran Paulsen, David George Behm, Vidar Andersen, Tom Erik Jorung Solstad, Olaf Prieske

**Affiliations:** 1 Department of Sport, Food and Natural Sciences, Western Norway University of Applied Sciences, Sogndal, Norway; 2 Department of Physical Performance, Norwegian School of Sport Sciences, Oslo, Norway; 3 School of Human Kinetics and Recreation, Memorial University of Newfoundland, St. John’s, NL Canada; 4 Division of Exercise and Movement, University of Applied Sciences for Sports and Management Potsdam, Olympischer Weg 7, Potsdam, 14471 Germany

## Abstract

**Background:**

Resistance training (RT) specificity has been confirmed for movement patterns (e.g., multi-joint or single joint), movement velocities, ranges of motion, and contraction types (e.g., dynamic vs isometric). However, a systematic analysis of the effects of dynamic mass-loaded (e.g., isoinertial) RT on dynamic versus isometric strength tests is lacking.

**Objective:**

We aimed to examine the specific effects of dynamic RT on dynamic (isoinertial) versus isometric muscle strength, including possible moderating factors (e.g., training length, single joint and multi-joint, upper body and lower body, RT status) and mechanisms (e.g., hypertrophy, muscle activation).

**Methods:**

A systematic literature search was conducted in MEDLINE (EBSCO), Web of Science, and Scopus up to March 2024. The included interventions contained at least ten training sessions, both dynamic and isometric muscle strength assessments before and after the training period, and healthy participants aged 16–60 years (encompassing untrained and trained individuals). Advanced RT approaches, such as electrical stimulation, isokinetic training, velocity-based training, and blood flow restriction training, were excluded. Within-subject, weighted standardized mean differences (SMDs) of the pre-intervention to post-intervention tests were calculated for both dynamic and isometric muscle strength measures using a random-effects model. Univariate sub-group analyses of RT status, intervention length, complexity (i.e., single-joint or multi-joint exercises), and body segments (i.e., upper and lower body) were independently computed. Random-effects meta-regressions were computed to examine if dynamic RT effects on dynamic and isometric muscle strength are predicted by RT effects on muscle hypertrophy or muscle activity.

**Results:**

Overall, 43 studies with 1660 participants across 72 different RT interventions were eligible for inclusion. The overall effect on dynamic strength was significant and moderate magnitude (SMD = 0.98, 95% confidence interval 0.91–1.06, *p* < 0.001), whereas the transfer to non-trained isometric strength measures was significant but small (SMD = 0.42, 95% confidence interval 0.35–0.49, *p* < 0.001). Sub-analyses demonstrated moderate-to-large task-specific effects (range SMD; 95% confidence interval 0.75–1.30) of conducting dynamic RT and only small-to-medium effects (range SMD; 0.29–0.70) of the transferability of muscle strength to the non-trained isometric contraction form. Muscle hypertrophy and activity changes did not significantly predict dynamic RT effects on dynamic and isometric muscle strength (*p* ≥ 0.222).

**Conclusions:**

Our findings demonstrated task specificity of dynamic RT, as dynamic strength increased with a two-fold larger effect size than non-trained isometric muscle strength. Medium-to-large effects were observed for the dynamic strength improvements in the different sub-group analyses with small-to-medium effects in the isometric improvements. The limited transferability of dynamic (task-specific) strength to non-trained isometric contractions suggests that these two strength outcomes represent different neuromuscular domains.

## Key Points

**Table:** 

Our findings indicate that the task specificity of dynamic contractions during resistance training revealed two-fold larger effect sizes for muscle strength than the transfer to non-trained isometric contractions.
Sub-group analyses of potential moderators (resistance training experience, multi-joint or single joint, intervention duration upper or lower body exercise, and position-matched multi-joint and single-joint exercises) displayed moderate-to-large effects of the task specificity dynamic improvement with only small-to-moderate effects of the transferability to non-trained isometric strength contraction.
Dynamic and isometric muscle strength gains were not predicted by muscle hypertrophy or changes in muscle activity in healthy adults.

## Introduction

Traditional resistance training (RT) typically uses multi-joint exercises such as squat, deadlift, and bench press. Traditional RT is isoinertial, defined by the constant external (mass) loading to motion [1], which will be referred to as dynamic strengthening exercises in this article. Task specificity refers to greater improvements in exercises used during the RT programs compared with the transferability of muscle strength and/or power to a non-trained exercise targeting the same muscle group [2, 3]. The principle of task specificity in RT had already been demonstrated in the 1950s [4]. Rasch and Morehouse [4] compared the effects of dynamic elbow flexion training on dynamic elbow flexor strength in a standing (i.e., trained) and supine (i.e., non-trained) position in healthy men. After a 6-week RT period, the participants demonstrated significantly larger strength improvements in the standing position when compared with the supine position. Subsequently, Thorstensson et al. [2] and Rutherford and Jones [5] demonstrated 3- to 11-fold greater improvements in trained dynamic isoinertial exercises compared with non-trained isometric contractions of the same muscle groups. Joint angle-specific RT adaptations were reported for the elbow flexors [6] and plantar flexors [7]. Several other studies from the 1970s to the 1990s reported velocity-specific RT adaptations with the greatest training gains at the training velocity and lower or trivial improvements at velocities above or below the RT velocity [8–11]. Hence, task specificity in RT has been demonstrated for movement pattern (multi-joint vs single-joint exercises), contraction forms, contraction velocities, contraction force, and joint angles [3, 12, 13]. Despite some recent meta-analyses addressing the generality of RT-induced strength gains and the transferability to other exercises (i.e., power clean’s impact on jump height performance) [3, 14], task specificity and transferability of muscle strength are frequently debated in the literature [15–18]. However, there appears to be considerable controversy regarding the external validity of isometric testing, especially examining the changes following dynamic RT programs and their relationship to dynamic muscle strength [19].

One repetition maximum (1RM) testing is the most frequently used approach to assess dynamic isoinertial strength [20] and has shown generally good-to-excellent test–retest reliability, regardless of number of familiarization sessions, RT experience, sex, age, exercise, and body segment [21]. Traditional 1RM testing (i.e., constant mass) requires less expensive equipment than other methods such as isokinetic or laboratory-based isometric testing, and is frequently used to describe and evaluate training effectiveness, as a valid measure of sport performance, and can be easily conducted in different populations (i.e., adolescents, athletes, general population, elderly individuals) [21, 22]. Still, isometric testing has been proven feasible [12, 15, 19], reliable [15], useful in rehabilitation settings [23, 24], helpful to examine force generation in specific joint angles [25–27], and associated with lower energy demands than dynamic testing [28, 29]. Furthermore, isometric muscle strength has been referred to as “pure” strength owing to the limited motor requirements of intramuscular and intermuscular coordination [30]. Therefore, isometric muscle strength may represent an individual’s ultimate strength capacity. Because of the limited proportionality between dynamic and isometric strength improvements, it has been suggested that these two strength qualities (dynamic and isometric) represent separate and specific adaptations referred to as neuromuscular domains [2, 5, 14, 15].

Even though dynamic RT and testing are more frequently conducted, isometric testing and training have demonstrated positive effects on sports-related skills (i.e., running, cycling, climbing) [31–33] and proven superior for improving angle-specific strength compared with dynamic training [27, 34]. Perhaps of most interest, isometric training may improve the force production at biomechanically disadvantageous joint positions [35–37]. However, isometric testing does not necessarily mimic daily tasks or sport performance, and dynamic strength (i.e., squat, bench or shoulder press) represents a more valid measure of daily life activities (e.g., chair rise, lifting objects) and sport performance (e.g., throwing, running, or jumping). In general, position-matched dynamic and isometric strength demonstrate < 50% shared variance [38, 39]. Because of the disproportionate transfer between dynamic and isometric strength performance [2, 3, 5], several authors have argued that these qualities of strength may be viewed as independent measurements of strength [19, 40]. In addition, most of the literature examining the association between these two contraction forms have used cross-sectional study designs [20, 41], which cannot necessarily be generalized to training responses. In daily life and sports, both dynamic and isometric strength are required while dynamic training interventions are most frequently conducted. The training-related transferability to non-trained contractions is therefore of relevance. Furthermore, the underlying mechanisms related to improving dynamic or isometric strength have frequently been debated [19, 40]. Recently, differences in muscle architectural gearing and shape have been demonstrated during dynamic and isometric contractions in a cross-sectional study [42] and may be proposed as one possible explanation of the lack of transferability of strength between the contraction forms. Still, longitudinal changes are missing in the literature. Other mechanisms include whether distinctive neuromuscular adaptations to dynamic RT (e.g., muscle activation pattern) transfer to isometric strength or if isometric strength needs to be trained independently. For example, it has been proposed that dynamic RT exercises (e.g., squats with free weights) are more dependent on neuromuscular adaptations, muscle coordination, and stability requirement owing to their complexity, rather than the muscle cross-sectional area, which has been associated with greater isometric strength [43–45]. Therefore, the aim of the present systematic review and meta-analysis was to analyze the task specificity of conducting dynamic RT for improving dynamic muscle strength and the transferability of dynamic strength gains to non-trained isometric muscle strength measures, potential moderating factors, and mechanisms (e.g., muscle hypertrophy and/or changes in muscle activity) among healthy adults. We hypothesize a greater dynamic strength improvement in the trained dynamic contraction form than in the isometric contraction form.

## Methods

This systematic review and meta-analysis was prospectively registered (PROSPERO CRD42023493208) and conducted in accordance with the Preferred Reporting Items for Systematic Reviews and Meta-Analyses (PRISMA) guidelines [46]. The present paper included one more sub-group analysis brought to our attention in the review process, which is not reported in the PROSPERO registration. We have included two sub-group analyses of position-matched exercises, i.e., studies that only tested multi-joints in both dynamic and isometric contraction forms and studies that only tested single joints in both dynamic and isometric contraction forms.

### Search Strategy

A systematic literature search was conducted between December 2023 and March 2024 and repeated in January 2025 in MEDLINE (EBSCO), Web of Science, and Scopus. The search strategy followed a Boolean approach using the operators “AND,” and “OR”: (((“Resistance trained” OR “healthy individuals” OR “recreational trained” OR “active”) AND (“resistance training” OR “strength training” OR “weight training” OR “dynamic training” OR “free-weight training”) AND (“muscle strength” OR “force output” OR “isometric” OR “repetition maximum” OR “torque” OR “maximal voluntary contraction”))). Additionally, we manually searched reference lists of included full-text studies and relevant review articles [3, 19, 47] for potentially eligible articles.

### Selection Criteria

**Fig. 1:**
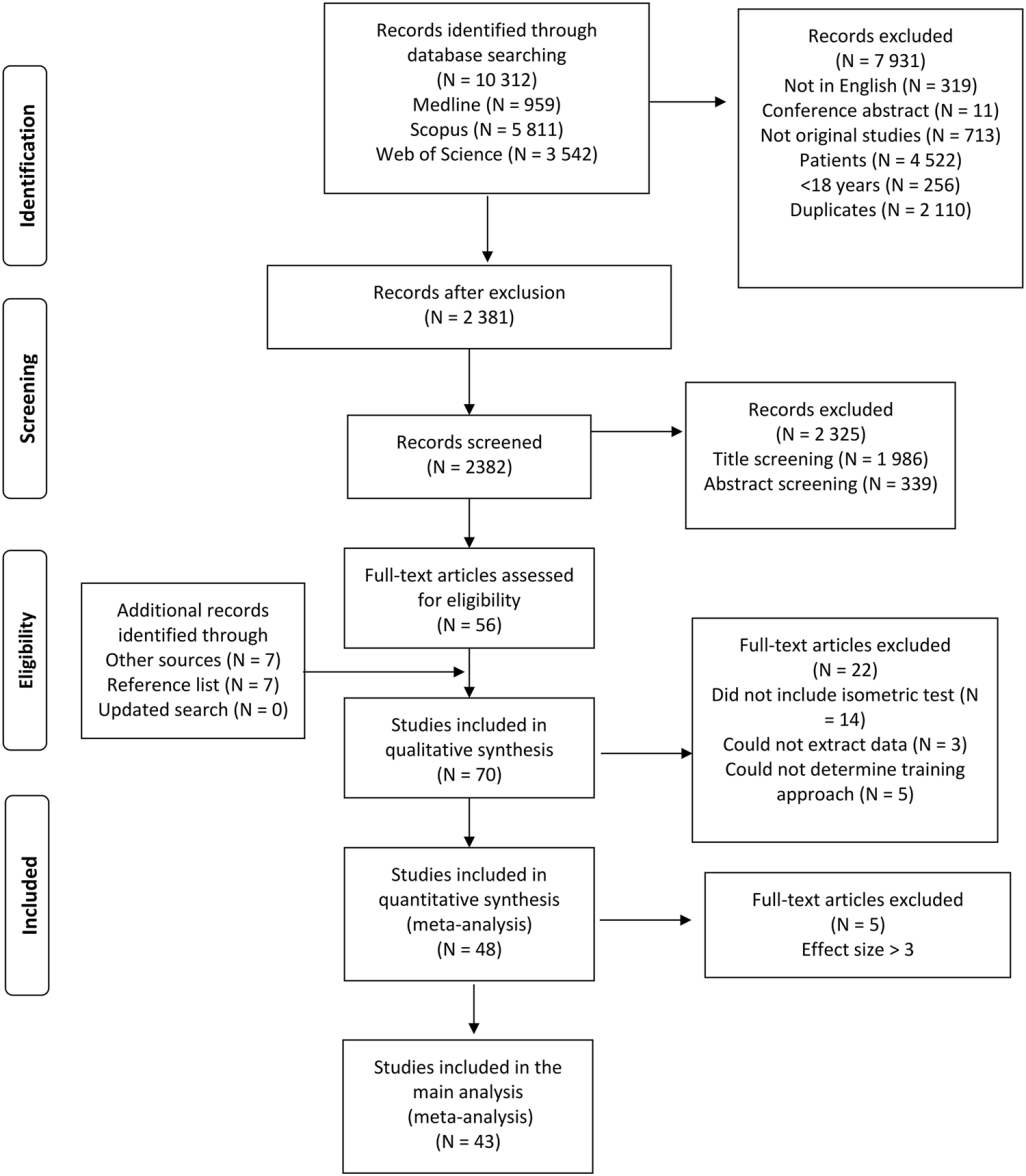
Flow chart illustrating the different phases of the study search and selection strategy

The search results were imported into Endnote (v.21; Thomson Reuters, Toronto, ON, Canada) for removal of duplications and sorting. Furthermore, the title and abstract screening was independently undertaken by AHS and VA, followed by full-text screening for eligibility (Fig. 1). To be included in this review, the article needed to meet the following inclusion criteria: (1) examine a traditional dynamic RT intervention using free weights or training machines; (2) report test data for both dynamic and isometric muscle strength of trained muscles/muscle groups; (3) report RT-related programming parameters (e.g., number of sets and/or repetitions); (4) include healthy participants (i.e., not patients) between 16 and 60 years; (5) include experimental designs (e.g., randomized controlled and experimental trials); (6) be published in a peer-reviewed journal; and (7) full text written in English language. There were no restrictions on publication year. Resistance training interventions including advanced RT approaches (e.g., electrical muscle stimulation, isokinetic training, instability RT, [accentuated] eccentric training, velocity-based training, blood-flow restriction training, see [48]) were excluded from the meta-analysis. Further, RT interventions conducted with fewer than ten training sessions or shorter than 5 weeks of duration were excluded. An overview of the included and excluded studies is presented in Fig. 1.

### Study Coding

Training duration (< 10 weeks or ≥ 10 weeks), RT status (sedentary, physically active, or RT trained), RT modality (single joint or multi-joint), and body segment (upper body or lower body) were used as potential moderators to examine the task specificity of dynamic RT on dynamic muscle strength and its transferability to non-trained isometric muscle strength. The RT status was classified based on the included prescriptions of the participants according to Peterson et al. [49]. If RT experience or RT status was not reported, but the participants were sport/physical education students or sports athletes, they were classified as physically active. Furthermore, a minimum of 6 months of systematic RT was used as the cut-off criterion for being classified as RT trained. Participants described as “not performed RT last year,” “healthy,” or “untrained” were classified as sedentary. In some of the included studies, whole-body RT programs were conducted, but muscle strength was assessed only in the lower or upper body parts. Based on the applied testing procedures, the included studies were classified as upper or lower body RT. Accordingly, the tested dynamic exercise was used to classify the included studies as an RT modality targeting multi-joints or single joints. All included studies were classified as a multi-joint or single-joint RT modality and used similar modalities during training and testing (i.e., tested and trained the same exercise).

### Data Extraction

A Microsoft Excel template used in previous systematic reviews and meta-analyses by our research group was used to extract the data. Data extraction included the dynamic and isometric tests being examined, the RT programming parameters (i.e., exercises, duration, weekly sessions, sets, repetitions, and training intensity), and the participants’ RT status, sample size, sex, and age. Two studies included dynamic and isometric tests of both the upper body and lower body [50, 51]. However, the majority of the included studies (35 out of 43 studies) examined the lower limbs and, therefore, only the lower body results in Bartolomei et al. [50] and Baker et al. [51] were included in the main analysis. If studies included several measurements of isometric conditions (e.g., a range of joint angles), the angle most frequently used in other eligible studies was selected. Furthermore, if a study compared several training groups meeting the inclusion criteria, all groups were included in the analyses. If studies reported multiple variables for the same outcome (e.g., several isometric force measurements at different joint angles in the full range of motion), a pre-defined ranking order was used to include only one outcome. In general, the most frequently reported outcome in the literature (i.e., a specific knee angle in isometric leg extension) was ranked higher than other angles. Accordingly, in studies reporting both 1RM and 10RM results, the 1RM was ranked higher than 10RM and used in the meta-analyses. Additionally, pre-test and post-test changes in dynamic and isometric muscle strength were extracted and used for further analysis. Means, standard deviations, and sample sizes were required for eligibility. If there were insufficient data to calculate an effect size, the corresponding authors were contacted for any missing data. If the corresponding author did not respond (two e-mails), the studies were excluded (*n* = 3).

When means and standard deviations were only presented in graph forms, the corresponding authors were contacted. In cases where the authors did not respond, a graph digitizer (Web Plot Digitizer) was used to estimate the values (*n* = 9). The Web Plot Digitizer has previously demonstrated high levels of intercoder reliability and validity for extracting data from graphs [52]. One author (AHS) extracted the data from the included studies, and a second author (NS) double-checked the data. All disagreements were resolved through personal communication between the two authors.

Studies reporting RT-induced muscle hypertrophy (e.g., magnetic resonance imaging or ultrasound measures) or muscle activity changes (e.g., M wave or root-mean square electromyography) were identified. The means, standard deviations, and sample sizes were used to calculate effect sizes (pre to post), which were used to examine if RT effects on dynamic and isometric strength were predicted by RT effects on hypertrophy and/or muscle activity.

### Study Quality

Two independent reviewers (AHS and NS) assessed the risk of bias and methodological quality using the screening scoring system for strength and conditioning-based training interventions of Brughelli et al. [53]. The scoring system included a combination of items from the Physical Evidence Database scale (PEDro) [54], Cochrane, and Delphi and assessed a 10-item scale (0–20 points). The scores for each of the ten items were rated as follows: 0 = clearly no/not reported; 1 = maybe; and 2 = clearly yes. The following factors were assessed: (1) clear inclusion criteria; (2) subjects were randomly allocated to groups; (3) clearly defined intervention; (4) groups were tested for similarity at baseline; (5) use of control groups; (6) outcome variables were clearly defined; (7) assessments were practically useful (i.e., valid, reliable); (8) duration of the intervention was practically useful; (9) appropriate between-group statistical analysis, and (10) point measures of variability. The included studies were interpreted as “poor quality,” “moderate quality,” “good quality,” and “excellent quality” with scores of < 10, 10–15, 16–19, and 20, respectively [55]. If there were any discrepancies in the given score, a third reviewer (VA) conducted an independent assessment on the scale to settle the disagreement. The overall score was not a criterion for inclusion or exclusion.

### Statistical Analysis

The software Review Manager (V.5.3.4; The Nordic Cochrane Centre, Copenhagen, Denmark, The Cochrane Collaboration, 2008) was used to conduct the meta-analysis. The within-subject standardized mean differences (SMDs) of both contraction forms (i.e., dynamic and isometric) were calculated [SMD = (mean post-test value − mean pre-test value)/standard deviation of mean pre-test value] [56–58]. Furthermore, the SMD was adjusted for sample size (i.e., Hedges’ *g*) using the factor [1 − (3/4 N-9)] with N being the total sample size [59]. Because of the independency of effect sizes, a two-level random-effects model was applied to weight each included study according to the magnitude of the respective standard error and to aggregate weighted mean-adjusted SMDs. The random-effects model was used as the relative weight assigned to each of the included studies has less impact on the computed combined effect size [60].

The level of between-study heterogeneity was assessed using *I*^2^ and chi-square statistics (*χ*^2^) to determine whether the results of the analyses were due to chance [61]. Low, moderate, and high heterogeneity (*I*^2^) corresponded to *I*^2^ of 25%, 50%, and 75% [62]. For the chi-square statistics (*χ*^2^), low *p*-values, or high *I*^2^ statistics, relative to the degrees of freedom (*df*) were observed to determine whether the results of the analysis were due to changes. A *p*-value of < 0.05 indicated a statistical significance. Furthermore, SMD values were classified as trivial, small, medium, or large, corresponding to values of < 0.2, 0.2–0.6, 0.6–1.2, and 1.2–2.0, respectively [63].

Further, random-effects meta-regressions were computed to examine if dynamic RT effects on dynamic and isometric muscle strength are predicted by RT effects on muscle hypertrophy or muscle activity. Therefore, the effects of dynamic RT on muscle hypertrophy and muscle activity were determined according to the formula introduced for the strength measures and included as potential predictors. In the regression models, the restricted maximum likelihood estimation method was applied and the Akaike information criterion for each model was calculated to directly compare the models. Regression analyses were performed using the open-source software JASP version 0.19.1 (University of Amsterdam, Amsterdam, The Netherlands).

## Results

### Study Characteristics

The search strategy initially identified 10,312 potential papers of which 48 studies [2, 16, 50, 51, 64–107] were finally included (Fig. 1). In the meta-analysis, six studies with 11 intervention groups were identified with an effect size greater than 3 [2, 16, 80, 84, 90, 103] (range 3.06–22.47) and were excluded as presumed outliers. However, one of the studies was included [80] in the main analyses as the large effect size was most likely a result of including healthy untrained subjects to a high-intensity RT program. The other outliers (effect size = 4.84–22.47) were removed from the main analyses [108]. The included 43 studies comprised 1660 participants across 72 different RT interventions (Table 1).

**Table 1:** Overview of the included studies

Study	Subjects included	Anthropometry(age, weight, height)	Fitness level	Dynamic test	Isometric test	RT intervention
Adamson et al. [63]	10 female	21 ± 3 years 68 ± 8 kg 171 ± 4 cm	Physically active	Unilateral arm flexion from supine position. 1RM test	Unilateral arm flexion at 100° (180° full arm extension). Peak force (N)	Unilateral arm flexion training. 8 weeks, 3 days·week^−1^, 5 sets of 5 reps (5RM load). Increased loads
Akagi et al. [64]	24 male	21 ± 2 years 64 ± 7 kg 174 ± 5 cm	No RT within last year	3RM in free-weight squat	Unilateral knee extension at 80° hip and 90° knee flexion. Peak torque (Nm)	Slow speed squats, 8 weeks, 3 days·week^−1^, 3 sets of 8 reps (40% of 1RM)
Alegre et al. [65]	16 male	22 ± 2 years 75 ± 9 kg 175 ± 8 cm	Physically active, not performed RT last year	Estimated 1RM in half squat using a 10RM test	Squat position at 90° knee angle for 4 s. Peak isometric force	Squats, 13 weeks, 3 days·week^−1^, 3–4 sets of 6–12 reps (50–60% of 1RM)
Andersen et al. INT 1 [105]	15 female	24 ± 6 years 67 ± 11 kg 169 ± 6 cm	RT trained (5 ± 4 years’ experience)	6RM in parallel squat	MVIC in unilateral leg extension with knee and hip angles of 90° for 2 s	Squats and split squat with elastic bands, 10 weeks, 2 days·week^−1^, 3 sets of 6–10 reps (6–10RM)
Andersen et al. INT 2 [105]	15 female	24 ± 4 years 67 ± 6 kg 166 ± 7 cm	RT trained (4 ± 4 years’ experience)	6RM in parallel squat	MVIC in unilateral leg extension with knee and hip angles of 90° for 2 s	Squats and split squat, 10 weeks, 2 days·week^−1^, 3 sets of 6–10 reps (6–10RM)
Baker et al. [50]	22 male	20 ± 3 years 75 ± 8 kg 180 ± 6 cm	Minimum of 6 months of RT	1RM in squat (no further details included)	Unilateral leg extension with knee and hip angles of 90° and 110°. Peak force output	Full body program, 12 weeks, 3 days·week^−1^, 3 sets of 6–10RM
Bartolomei et al. INT 1 [49]	10 male	21.1 ± 4 years 79 ± 11 kg 177 ± 4 cm	Resistance trained (6.6 ± 3.5 years’ experience)	1RM in squat (trochanter at the same level as the knee)	Isometric squat at a knee and hip angle of 90° for 6 s	Total body program, 10 weeks, 3 days·week^−1^, 5 sets of 6 reps (1 RIR)
Bartolomei et al. INT 2 [49]	11 male	25 ± 4 years 79 ± 10 kg 175 ± 6 cm	Resistance trained (6.6 ± 3.5 years’ experience)	1RM in squat (trochanter at the same level as the knee)	Isometric squat at a knee and hip angle of 90° for 6 s	Split program, 10 weeks, 3 days·week^-1^, 5 sets of 6 reps (1 RIR)
Beyer et al. [66]	9 male	23 ± 3 years 77 ± 14 kg 174 ± 8 cm	No RT within last year	1RM in unilateral leg press (no further details included)	Peak force of a 5-s isometric knee extension at a hip and knee angle of 110° (180° = full extension)	Total body program, 4 weeks, 3 days·week^−1^, 3 sets of 8–10 reps (80% of 1RM)
Bloomquist et al. INT 1 [67]	9 male	23 ± 3 years 80 ± 15 kg 179 ± 6 cm	Not performed squat more than 1 per week last 6 months	1RM in squat (60° knee flexion)	Peak torque of a 5-s isometric knee extension at knee angle of 75° (0° = full knee extension)	Barbell free weight squat 60° knee flexion, periodized over 12 weeks, 3 days·week^−1^, 3–5 sets of 3–10 reps (3–10RM)
Bloomquist et al. INT 2 [67]	8 male	25 ± 6 years 79 ± 6 kg 181 ± 5 cm	Not performed squat more than 1 per week last 6 months	1RM in squat (60° knee flexion)	Peak torque of a 5-s isometric knee extension at knee angle of 75° (0° = full knee extension)	Barbell free weight squat 120° knee flexion, periodized over 12 weeks, 3 days·week^−1^, 3–5 sets of 3–10 reps (3–10RM)
Botton et al. INT 1 [68]	14 female	25 ± 1 years 61 ± 6 kg 163 ± 7 cm	Not performed systematic RT last 3 months	1RM in bilateral knee extension (90° of knee flexion, 0° = full knee extension)	Peak torque of a 5-s isometric knee extension at 60° knee angle (0° = full knee extension)	Unilateral knee extension training, periodized over 12 weeks, 2 days·week^−1^, 2–4 sets of 5–15 reps (5–15 RM) + full body program
Botton et al. INT 2 [68]	15 female	24 ± 4 years 57 ± 5 kg 160 ± 6 cm	Not performed systematic RT last 3 months	1RM in bilateral knee extension (90° of knee flexion, 0° = full knee extension)	Peak torque of a 5-s isometric knee extension at 60° knee angle (0° = full knee extension)	Bilateral knee extension training, periodized over 12 weeks, 2 days·week^−1^, 2–4 sets of 5–15 reps (5–15RM) + full body program
Braith et al. INT 1 [69]	Mix 13 male 12 female	23–29 years 57–83 kg 165–178 cm	Not performed RT last 12 months	Training load in bilateral Nautilus knee extension with fatigue between 7 and 10 reps	Maximal peak torque of 1- to 2-s isometric knee extension obtained from the 70°–171° knee angles (0° = full knee extension)	Bilateral knee extension training (Nautilus), 10 weeks, 2 days·week^−1^, 1 sets of 7–10 reps (7–10RM)
Braith et al. INT 2 [69]	Mix 9 male 10 female	25–29 years 61–73 kg 163–180 cm	Not performed RT last 12 months	Training load in bilateral Nautilus knee extension with fatigue between 7 and 10 reps	Maximal peak torque of 1- to 2-s isometric knee extension obtained from the 70°–171° knee angles (0° = full knee extension)	Bilateral knee extension training (Nautilus), 10 weeks, 3 days·week^−1^, 1 sets of 7–10 reps (7–10RM)
Braith et al. INT 3 [69]	Mix 13 male 12 female	24–25 years 61–77 kg 165–180 cm	Not performed RT last 12 months	Training load in bilateral Nautilus knee extension with fatigue between 7 and 10 reps	Maximal peak torque of 1- to 2-s isometric knee extension obtained from the 70°–171° knee angles (0° = full knee extension)	Bilateral knee extension training (Nautilus), 18 weeks, 2 days·week^−1^, 1 sets of 7–10 reps (7–10RM)
Braith et al. INT 4 [69]	Mix 12 male 10 female	24–26 years 64–77 kg 167–182 cm	Not performed RT last 12 months	Training load in bilateral Nautilus knee extension with fatigue between 7 and 10 reps	Maximal peak torque of 1- to 2-s isometric knee extension obtained from the 70°–171° knee angles (0° = full knee extension)	Bilateral knee extension training (Nautilus), 18 weeks, 3 days·week^−1^, 1 sets of 7–10 reps (7–10RM)
Chaouachi et al. [70]	15 male	19 ± 1 years 70 ± 5 kg 178 ± 6 cm	Healthy adults not attending any training programs	1RM in unilateral full ROM chest press (90°–10° elbow angle (0° full extension)	Unilateral isometric elbow flexion at 90° (3–5 s)	Unilateral chest press, 8 weeks, 3 days·week^−1^, 3–4 sets of 6–10 reps (6–10RM)
Colomer-Poveda et al. INT 1 [71]	11 male	22 ± 1 years	Recreationally active not performing RT	1RM in unilateral full ROM (90°–180°) knee extension	Unilateral 3- to 5-s MVIC with a hip and knee angle of 90°	Unilateral knee extension, 4 weeks, 4 days·week^−1^, 6 sets of 5–6 reps (75% of 1RM)
Colomer-Poveda et al. INT 2 [71]	11 male	21 ± 1 years	Recreationally active not performing RT	1RM in unilateral full ROM (90°–180°) knee extension	Unilateral 3- to 5-s MVIC with a hip and knee angle of 90°	Unilateral knee extension, 4 weeks, 4 days·week^−1^, 3 sets to failure (75% of 1RM)
Comfort et al. INT 1 [72]	Mix 12 male 4 female	19 ± 2 years 71 ± 12 kg 179 ± 8 cm	Professional soccer players and Collegiate athletes	1RM power clean (NSCA standardized protocol)	Peak isometric mid-thigh pull force with a knee and hip angle of 133° and 146°	Lower-body RT program including the catch phase in power clean, 2 × 4 weeks mesocycle, 2 days·week^−1^, 3 sets of 3 reps (55–82.5% of 1RM)
Comfort et al. INT 2 [72]	Mix 14 male 4 female	20 ± 3 years 66 ± 10 kg 173 ± 10 cm	Professional soccer players and collegiate athletes	1RM power clean (NSCA standardized protocol)	Peak isometric mid-thigh pull force with a knee and hip angle of 133° and 146°	Lower-body RT program excluding the catch phase in power clean, 2 × 4 weeks mesocycle, 2 days·week^−1^, 3 sets of 5 reps (55–82.5% of 1RM)
Contreras et al. INT 1 [73]	14 subjects (sex n.a.)	15.5 ± 1 years 78 ± 12 kg 179 ± 5 cm	Adolescent rugby and rowing athletes with 1 year squat experience	3RM in front squat (thigh parallel with floor)	Peak isometric mid-thigh pull force during 5 s with barbell locked at mid-thigh position	Periodized upper body + hip trust RT program, 6 week, 2 days·week^−1^, 4 sets of 6–12 reps (6–12RM)
Contreras et al. INT 2 [73]	14 subjects (sex n.a.)	15.5 ± 1 years 81 ± 12 kg 182 ± 6 kg	Adolescent rugby and rowing athletes with 1 year squat experience	3RM in hip trust	Peak isometric mid-thigh pull force during 5 s with barbell locked at mid-thigh position	Periodized upper body + squat RT program, 6 week, 2 days·week^−1^, 4 sets of 6–12 reps (6–12RM)
Cook et al. [74]	Mix 3 male 3 female	19 and 21 years 75 and 55 kg 173 and 162 cm	Not performed RT last 3 months	1RM in bilateral seated leg press	Peak torque of unilateral knee extensors with a hip and knee angle of 85° and 60°	6 week leg press, and knee extension RT, 3 days·week^−1^, 3 sets of 10 reps (70% of 1RM)
Cormie et al. [75]	8 male	21 ± 1 years 80 ± 15 kg 175 ± 9 cm	Recreationally trained (1.5 ± 0.3 in squat-to-weight ratio)	1RM in squat (no further details included)	Isometric peak force in squat at a 100° knee angle	12 week of strength-power squat jump, 5 sets of 6 reps (30% of 1RM) and squat training, 3 sets of 3 reps (90% of 1RM), 2 days·week^−1^
Diniz et al. INT 1 [76]	10 female	22 ± 3 years 60 ± 8 kg 162 ± 5 cm	Untrained	1RM in knee extension (hip angle of 110°)	Peak torque in knee extension at 60° knee angle (0° = full knee extension)	Periodized 10-week knee extension training, 3 days·week^−1^, 3–5 sets of 6 reps (50% 1RM) using 5-s concentric and eccentric 1-s phases
Diniz et al. INT 2 [76]	10 female	22 ± 3 years 60 ± 8 kg 162 ± 5 cm	Untrained	1RM in knee extension (hip angle of 110°)	Peak torque in knee extension at 60° knee angle (0° = full knee extension)	Periodized 10-week knee extension training, 3 days·week^−1^, 3–5 sets of 6 reps (50% 1RM) using 3-s concentric and 3-s eccentric phases
Diniz et al. INT 3 [76]	10 female	22 ± 3 years 60 ± 8 kg 162 ± 5 cm	Untrained	1RM in knee extension (hip angle of 110°)	Peak torque in knee extension at 60° knee angle (0° = full knee extension)	Periodized 10-week knee extension training, 3 days·week^−1^, 3–5 sets of 6 reps (50% 1RM) using 1-s concentric and 5-s eccentric phases
Drummond et al. [77]	10 male	22 ± 3 years 72 ± 11 kg 174 ± 7 cm	Recreationally active with no RT experience	1RM in elbow flexion (Scott bench)	Peak force in unilateral elbow flexion at 90° elbow angle (6-s contraction)	12-week unilateral elbow flexion training, 3 days·week^−1^, 4 sets of 8–10 reps (8–10RM)
Erskine et al. [78]	33 male	23 ± 3 years 75 ± 11 kg 176 ± 6 cm	Not performed RT last 12 months	1RM in elbow flexion (modified preacher bench)	MVIC in elbow flexion at 60° elbow angle (0° = full elbow extension) lasting 3 s	Periodized 12-week elbow flexion training, 3 days·week^−1^, 2–4 sets of 8–10 reps (8–10RM)
Erskine et al. [79]	53 male	20 ± 3 years 77 ± 11 kg 178 ± 5 cm	Not performed RT last 12 months	1RM knee extension	Peak torque leg extension at 70°–90° knee angle (2–3 s MVIC)	Periodized 9-week unilateral knee extension training, 3 days·week^−1^, 4 sets of 10 reps (80% of 1RM)
Faude et al. [80]	8 male	23 ± 3 years 79 ± 5 kg 176 ± 5 cm	Third-level Swiss football league	1RM half squat (maximal knee angle at 100°)	Isometric peak force in leg press (5-s MVC) at 100° knee and 90° hip angles	7-week strength and power RT, 2 days·week^−1^, 4 sets of 4 reps (50–60% and 90% of 1RM)
Fisher et al. INT 1 [101]	12 male	27 ± 7 years 82 ± 8 kg 179 ± 5 cm	Resistance trained (> 2 years RT)	1RM Romanian deadlift	Isometric peak torque in hip extension at 36° hip flexion (72° = full lumbar flexion)	10-week RT, 1 day·week^−1^, 1 sets of 8–12 reps (80% of 1RM)
Fisher et al. INT 2 [101]	12 male	27 ± 7 years 82 ± 8 kg 179 ± 5 cm	Resistance trained (> 2 years RT)	1RM lumbar extension machine	Isometric peak torque in hip extension at 36° hip flexion (72° = full lumbar flexion)	10-week RT, 1 day·week^−1^, 1 sets of 8–12 reps (80% of 1RM)
Fimland et al. [81]	10 male	23 ± 3 years 80 ± 7 kg 184 ± 6 cm	Experienced in RT, but systematically RT	1RM leg press from 0 to 90° knee angle (0° = full extended)	MVIC in planter flexion (90°, 80° and 90° angle at ankle, knee, and hip)	8-week leg press RT, 3 days·week^−1^, 4 sets of 4 reps (85–90% of 1RM). Concentric movement ending in a plantar flexion
Fry et al. INT 1 [82]	7 male	22 ± 3 years 81 ± 15 kg 179 ± 9 cm	Not performed RT last 12 months	10RM parallel barbell squat (hip and knee angle < 90°)	MVIC torque in a 5-s leg extension at 60° knee angle (0° = fully extended)	8-week squat RT, 3 days·week^−1^, 3 sets of 10 reps (10RM)
Fry et al. INT 2 [82]	7 male	21 ± 4 years 78 ± 8 kg 178 ± 8 cm	Not performed RT last 12 months	10RM in bilateral leg extension	MVIC torque in a 5-s leg extension at 60° knee angle (0° = fully extended)	8-week leg extension RT, 3 days·week^−1^, 1 sets of 7–10 reps (7–10RM)
Graves et al. INT 1 [83]	Mix 13 male 8 female	35 ± 7 years 77 ± 18 kg 174 ± 11 cm	No RT experience	Training load in leg extension machine (8–12RM) limited to 72° lumbar extension	MVIC hip torque at 36° of lumbar flexion	12 week lumbar extension RT with pelvic stabilization, 1 day·week^−1^, 1 set of 8–12 reps (8–12RM) with 2-s concentric and 4-s eccentric contraction
Graves et al. INT 2 [83]	Mix 24 male 17 female	32 ± 11 years 73 ± 14 kg 173 ± 11 cm	No RT experience	Training load in leg extension machine (8–12RM) no limited lumbar extension	MVIC hip torque at 36° of lumbar flexion	12-week lumbar extension RT with pelvic stabilization, 1 day·week^−1^, 1 set of 8–12 reps (8–12RM) with 2-s concentric and 4-s eccentric contraction
Hubal et al. [103]	Mix 243 male 342 female	24 ± 0.2 years 70 ± 0.7 kg 170 ± 0.5 cm	Not performed RT last 12 months	1RM in unilateral elbow flexion	MVIC elbow flexion at 90°	12-week elbow flexion and elbow extension RT with progression, 45–60 min sessions, 3 sets of 6–12 reps (6–12RM)
Lamas et al. [85]	14 male	23 ± 4 years 76 ± 9 kg 177 ± 6 cm	Minimum 2 years of RT experience	1RM in squat with proper technique (no further details were included)	MVIC in a horizontal leg press device at 100° and 95° hip and knee angles	Periodized 8-week RT, 3 days·week^−1^, 1–4 sets of 4–10 reps (4–10RM)
Marshall et al. INT 1 [106]	11 male	26 ± 1 years 80 ± 3 kg 177 ± 2 cm	Resistance trained (6.6 RT experience)	1RM in squat (90° knee flexion)	Peak torque in knee extension at 70° (0° fully extended) in 3 s	6-week squat (90° knee flexion) RT, 2 days·week^−1^, 1 set to failure (80% of 1RM)
Marshall et al. INT 2 [106]	11 male	31 ± 3 years 85 ± 4 kg 178 ± 2 cm	Resistance trained (6.6 RT experience)	1RM in squat (90° knee flexion)	Peak torque in knee extension at 70° (0° fully extended) in 3 s	6-week squat (90° knee flexion) RT, 2 days·week^−1^, 4 sets to failure (80% of 1RM)
Marshall et al. INT 3 [106]	10 male	26 ± 2 years 85 ± 6 kg 169 ± 8 cm	Resistance trained (6.6 RT experience)	1RM in squat (90° knee flexion)	Peak torque in knee extension at 70° (0° fully extended) in 3 s	6-week squat (90° knee flexion) RT, 2 days·week^−1^, 8 sets to failure (80% of 1RM)
Mattocks et al. INT 1 [100]	Mix 7 male 11 female	21 ± 3 years 79 ± 23 kg 179 ± 2 cm	Not performed RT last 6 months	1RM in knee extension (no further details were included)	Peak torque in knee extension at 90° knee flexion	8-week knee extension RT, 2 days·week^−1^, 4 sets to failure (8–12RM)
Mattocks et al. INT 2 [100]	Mix 10 male 10 female	22 ± 4 years 70 ± 14 kg 174 ± 9 cm	Not performed RT last 6 months	1RM in knee extension (no further details were included)	Peak torque in knee extension at 90° knee flexion	8-week knee extension RT, 2 days·week^−1^, 5 attempts to reach 100% of 1RM
Ogasawara et al. INT 1 [86]	7 male	25 ± 3 years 65 ± 6 kg 173 ± 8 cm	Not performed RT last 2 years	1RM in bench press with grip width at 200% of biacromial breadth	MVIC in a seated chest press at 90° elbow angle (0° fully extended)	24-week bench press RT, 3 days·week^−1^, 3 sets of 10 reps (75% of 1RM)
Ogasawara et al. INT 2 [86]		24 ± 2 years 70 ± 13 kg 170 ± 3 cm	Not performed RT last 2 years	1RM in bench press with grip width at 200% of biacromial breadth	MVIC of the elbow extensors at an 90° elbow angle (0° fully extended)	24-week bench press RT divided in 3 training cycles, 3 days·week^−1^, 3 set of 10 reps (75% of 1RM)
Painter et al. INT 1 [87]	Mix *n* = 14	20 ± 1 years 86 ± 31 kg 177 ± 11 cm	Track and field athletes	1RM parallel squat (no further details included)	Isometric mid-thigh pull (no further details included)	10-week full body RT, 3 days·week^−1^, 3–5 sets of 3–10 reps (65–80% of 1RM)
Painter et al. INT 2 [87]	Mix *n* = 12	19 ± 1 years 81 ± 18 kg 177 ± 6 cm	Track and field athletes	1RM parallel squat (no further details included)	Isometric mid-thigh pull (no further details included)	10-weeks full body RT, 3 days·week^−1^, 3 sets of 3–12 reps (3–12RM)
Paulsen et al. INT 1 [104]	10 male	20–30 years 182 ± 5 cm	No RT experience	1RM squat (no further details included)	MVIC in knee extension (60° from full knee extension)	6-weeks full body RT, 3 days·week^−1^, 1 set of 7 reps (7RM)
Paulsen et al. INT 2 [104]	8 male	20–30 years 182 ± 5 cm	No RT experience	1RM squat (no further details included)	MVIC in knee extension (60° from full knee extension)	6-week full body RT, 3 days·week^−1^, 3 sets of 7 reps (7RM)
Saeterbakken et al. INT 1 [88]	13 male	23 ± 5 years 76 ± 7 kg 178 ± 7 cm	RT experienced (5 ± 4 years)	10RM in squat (90° knee flexion) in a Smith machine	MVIC in unilateral knee extension (90° knee flexion). Mean force in 2 s	Periodized 10-week squat RT in a Smith machine, 2 days·week^−1^, 3–4 sets of 6–10 reps (6–10RM)
Saeterbakken et al. INT 2 [88]	11 male	23 ± 2 years 82 ± 9 kg 183 ± 4 cm	RT experienced (5 ± 4 years)	10RM in squat (90° knee flexion) in a Smith machine	MVIC in unilateral knee extension (90° knee flexion). Mean force in 2 s	Periodized 10-week free weight squat RT, 2 days·week^−1^, 3–4 set of 6–10 reps (6–10RM)
Stien et al. INT 1 [90]	20 female	22 ± 1 years 65 ± 5 kg 167 ± 3 cm	Approximately 1 years of RT experience	6RM in leg press at 90° knee flexion and 70° hip angle (0° fully extended)	MVIC over a 3-s window in knee extension at 90° in knee and hip joints (0° fully extended)	Periodized 8-week leg press RT, 2–3 days·week^−1^, 3–4 sets of 6–10 reps (6–10RM)
Stien et al. INT 2 [90]	18 female	23 ± 1 years 65 ± 5 kg 166 ± 3 cm	Approximately 1 years of RT experience	6RM in leg extension at 90° in knee and hip joints (0° fully extended)	MVIC over a 3-s window in knee extension at 90° in knee and hip joints (0° fully extended)	Periodized 8-week leg extension and kick back RT, 2–3 days·week^−1^, 3–4 sets of 6–10 reps (6–10RM)
Suchomel et al. INT 1 [91]	9 male	23 ± 4 years 86 ± 13 kg 181 ± 6 cm	Resistance trained (relative 1RM squat = 1.75)	1RM in power clean	Isometric mid-thigh pull at knee and hip angles of 125°–135° and 140°–150°	Periodized 10-week RT program + power clean RT with the catch phase 3 days·week^−1^, 3–5 sets of 2–10 reps (50–95% of 1RM)
Suchomel et al. INT 2 [91]	9 male	22 ± 2 years 84 ± 17 kg 180 ± 4 cm	Resistance trained (relative 1RM squat = 1.73)	1RM in power clean	Isometric mid-thigh pull at knee and hip angles of 125°–135° and 140°–150°	Periodized 10-week RT program + power clean without the pull phase, 3 days·week^−1^, 3–5 sets of 2–10 reps (50–95% of 1RM)
Suchomel et al. INT 3 [91]	9 male	22 ± 1 years 83 ± 14 kg 173 ± 9 cm	Resistance trained (relative 1RM squat = 1.76)	1RM in power clean	Isometric mid-thigh pull at knee and hip angles of 125°–135° and 140°–150°	Periodized 10-week RT program + power clean with overload stimulus, 3 days·week^−1^, 3–5 sets of 2–10 reps (75–35% of 1RM)
Toohey et al. INT 1 [92]	12 female	All subjects 20 ± 1 years 68 ± 7 kg 171 ± 7 cm	Division 1 volleyball and football players	1RM in squat with proper technique and meeting ROM criteria	Peak force in isometric mid-thigh pull with barbell position mid- thigh	10-week full body RT program with probiotic supplementation, 3 days·week^−1^, 3–4 sets of 3–10 reps (3–10RM)
Toohey et al. INT 2 [92]	11 female	All subjects 20 ± 1 years 68 ± 7 kg 171 ± 7 cm	Division 1 volleyball and football players	1RM in squat with proper technique and meeting ROM criteria	Peak force in isometric mid-thigh pull with barbell position mid-thigh	10-week full body RT program, 3–4 days·week^−1^, 3–4 sets of 3–10 reps (3–10RM)
Travis et al. INT 1 [93]	Mix 14 male 2 female	All subjects 24 ± 4 years 90 ± 21 kg 174 ± 8 cm	Powerlifters (relative 1RM squat = 2.0)	1RM in squat according to powerlifting rules	Peak force in isometric squat position at 90° knee angle	6-week full body step taper RT program, 3 days·week^−1^, 4 sets of 3–5 reps (85% of 1RM)
Travis et al. INT 2 [93]	Mix 14 male 2 female	All subjects 24 ± 4 years 90 ± 21 kg 174 ± 8 cm	Powerlifters (relative 1RM squat = 2.0)	1RM in squat according to powerlifting rules	Peak force in isometric squat position at 90° knee angle	6-week full body exponential taper RT program, 3 days·week^−1^, 4 sets of 3–5 reps (85% of 1RM)
Trzaskoma et al. INT 1 [94]	9 male	22 ± 2 years 77 ± 7 kg 180 ± 7 cm	RT experienced	1RM in squat at 90° knee flexion	Sum of maximal torque of hip and knee flexor and extensor at 90° knee and hip angle. 5-s contraction time	3-week full body RT program, 4 days·week^−1^, 3–4 sets of 3–12 reps (65–125% of 1RM)
Trzaskoma et al. INT 2 [94]	9 male	23 ± 1 years 78 ± 9 kg 181 ± 5 cm	RT experienced	1RM in squat at 90° knee flexion	Sum of maximal torque of hip and knee flexor and extensor at 90° knee and hip angle. 5-s contraction time	3-week full body RT program, 4 days·week^−1^, 4–6 sets of 6–12 reps (80–135% of 1RM)
Weir et al. [95]	9 male	24 ± 3 years 85 ± 8 kg 77 ± 12 cm	Not performed RT last 6 months	1RM eccentric leg extension (1–2 s lowering the weight)	MVIC knee extension at 75° knee angle below the horizontal plane	Periodized 8-week leg extension eccentric RT program, 3 days·week^−1^, 3–5 sets of 6 reps (6RM)
Wilson et al. [96]	13 Sex not reported	22 ± 4 years 69 ± 9 kg 173 ± 9 cm	Conducting RT last 12 months (half-squat strength > body weight)	Isokinetic peak torque in leg extension at an angular velocity of 5.2 rad s^−1^	Isometric squat at 2.36 rad s (135°) knee angle	10-weeks squat RT program, 2 days·week^−1^, 3–6 sets of 6–10 reps (6–10RM)
Wirth et al. INT 1 [97]	43 male	24 ± 3 years 82 ± 10 kg 182 ± 8 cm	Students (no further details were reported)	1RM in parallel squat at 60°–70° knee angle	MVIC in legwork machine (hip and knee angle at 60° and 120°	8-week squat RT program, 2 days·week^−1^, 3–6 sets of 6–10 reps (6–10RM)
Wirth et al. INT 2 [97]	40 male	24 ± 2 years 81 ± 8 kg 180 ± 7 cm	Students (no further details were reported)	1RM in leg press at 90° knee angle	MVIC in legwork machine (hip and knee angle at 60° and 120°)	Periodized 8-week leg press RT program, 2 days·week^−1^, 5 sets of 4–10 reps (8–10RM)
Wirth et al. [98]	15 male	24 ± 2 years 76 ± 7 kg 180 ± 3 cm	Little or no RT experience	1RM in unilateral leg press (no further details were included)	MVIC in legwork machine (hip and knee angle at 60° and 120°)	6-week eccentric leg press RT program, 3 days·week^−1^, 5 sets of 3 reps (3RM)
Yasuda et al. [99]	10 male	25 ± 3 years 63 ± 9 kg 172 ± 5 cm	None to moderate RT experience (9 out of 40 had RT experience)	1RM in free-weight flat bench press	MVIC torque (3-s window) in elbow extensors at 90° elbow angle (0° fully extended)	6-week bench press RT program, 3 days·week^−1^, 3 sets of 10 reps (75% of 1RM)

*INT* intervention, *MVIC* maximal voluntary isometric contraction, *n.a.*  not available,* NSCA* National Strength and Conditioning Association, *reps* repetitions, *RM* repetition maximum, *ROM* range of motion, *RPE* rate of perceived exertion, *RM* repetition maximum,* RT* resistance training, *NSCA* National Strength and Conditioning Association, *RPE* rate of perceived exertion, *MVIC* maximal voluntary isometric contraction

For the sub-group analysis according to RT status, 29 interventions included sedentary participants, 24 interventions included physically active participants, and 16 interventions reported participants with more than 6 months of RT experience. In terms of RT modalities, 44 intervention studies and 24 intervention studies were categorized as multi-joint and single joint, respectively. In terms of RT duration, 34 interventions lasted less than 10 weeks and 36 interventions had a RT duration of more than 10 weeks. Finally, eight interventions examined upper body exercises, whereas 65 interventions examined lower body exercises.

Of the included studies, 13 studies [50, 66–69, 75, 79, 80, 87, 93, 94, 100, 101] reported muscle hypertrophy using either ultrasound or magnetic resonance imaging of the morphological adaptions. Accordingly, nine studies [67, 69, 72, 79, 80, 89, 91, 106] reported muscle activity using electromyography (M wave or root-mean-square) of neurological adaptations.

### Outcome of Assessment of Reporting Quality

Study quality assessment scores ranged from 14 to 20 on the 0–20 point scale (median 17, 25th to 75th percentiles ± 1). The score indicated a good-to-excellent methodological quality (moderate quality *n* = 6, good quality *n* = 36, and excellent quality *n* = 1) of the included studies. Of note, the most common criterion, which was not met, was the inclusion of a control group, which was not considered for the present research question.

### Meta-analyses

**Fig. 2:**
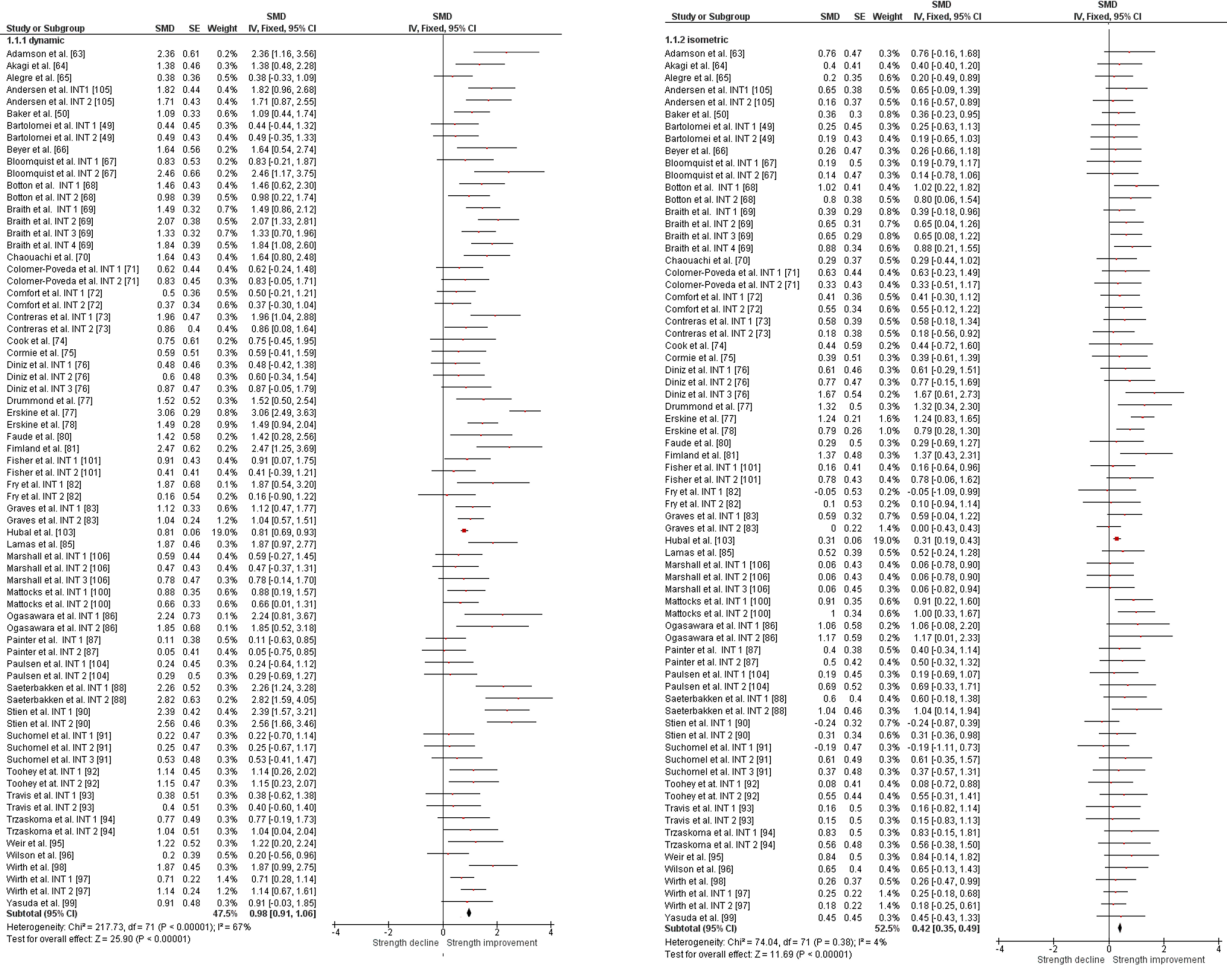
Standardized mean difference (SMD) of conducting dynamic resistance training on task-specificity (dynamic) strength and the transferability of strength (isometric). *CI* confidence interval, *df* degrees of freedom, *INT* intervention, *IV* inverse variance, *Random* random-effects model, *SE* standard error

**Fig. 3:**
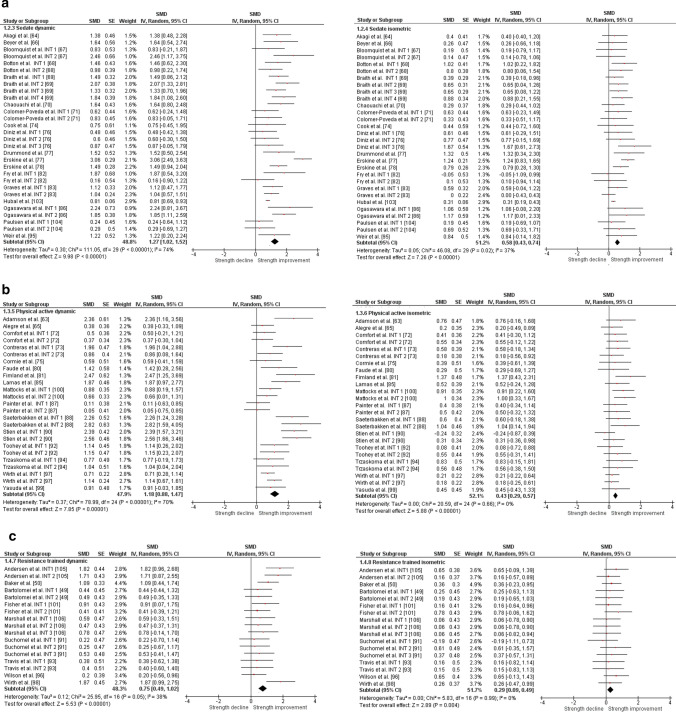
Standardized mean difference (SMD) of conducting dynamic resistance training on task-specificity (dynamic) strength and the transferability of strength (isometric) among **a** sedentary subjects, **b** physically active subjects, and **c** resistance-trained subjects. *CI* confidence interval, *df* degrees of freedom, *INT* intervention, *IV* inverse variance, *Random* random-effects model, *SE* standard error, *SMD* standardized mean difference

**Fig. 4:**
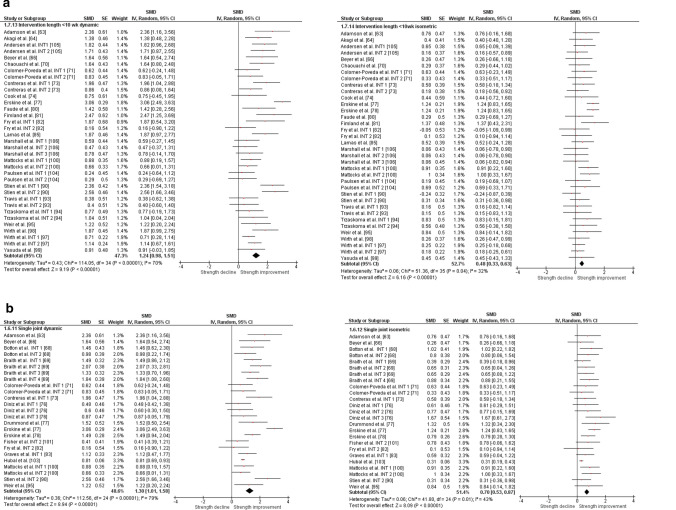
Standardized mean difference (SMD) of conducting dynamic RT on task-specificity (dynamic) strength and the transferability of strength (isometric) for **a** multi-joint tested exercises and **b** single-joint exercises. *CI* confidence interval, *df* degrees of freedom, *INT* intervention, *IV* inverse variance, *Random* random-effects model, *SE* standard error

**Fig. 5:**
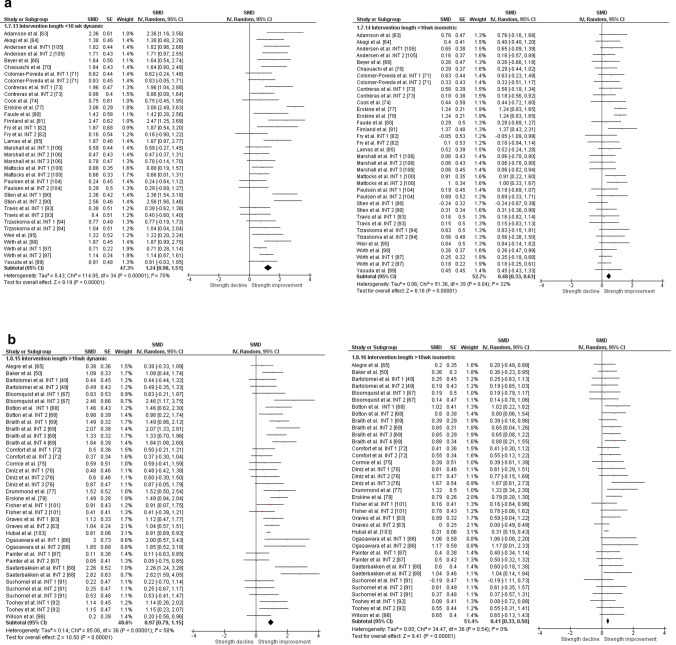
Standardized mean difference (SMD) of conducting dynamic resistance training on task-specificity (dynamic) strength and the transferability of strength (isometric) for intervention length **a** shorter than 10 weeks and **b** 10 weeks or longer. *CI* confidence interval, *df* degrees of freedom, *INT* intervention, *IV* inverse variance, *Random* random-effects model, *SE* standard error

**Fig. 6:**
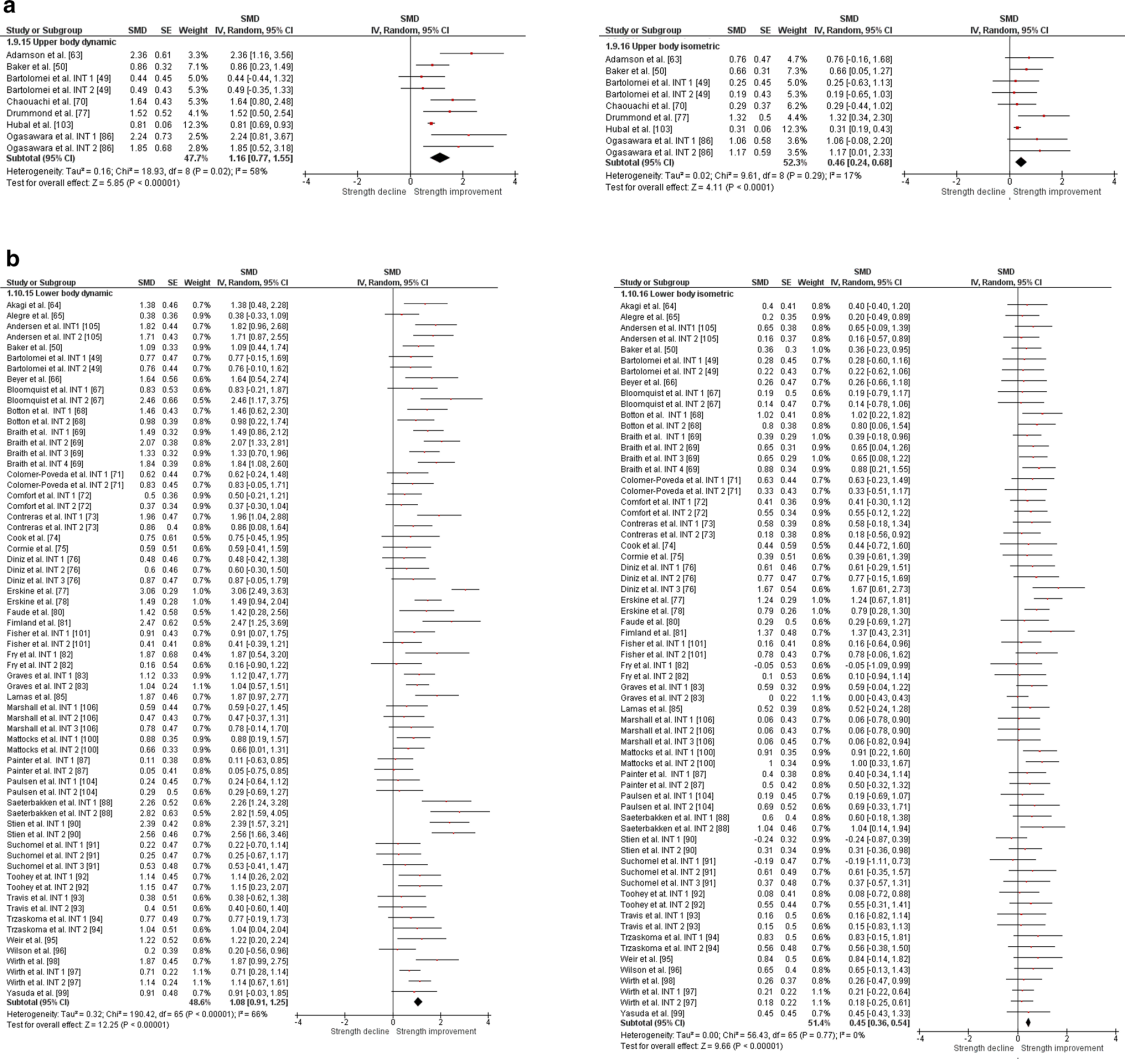
Standardized mean difference (SMD) of conducting dynamic resistance training on task-specificity (dynamic) strength and the transferability of strength (isometric) for **a** upper body and **b** lower body. *CI* confidence interval, *df* degrees of freedom, *INT* intervention, *IV* inverse variance. *Random* random-effects model, *SE* standard error

**Fig. 7:**
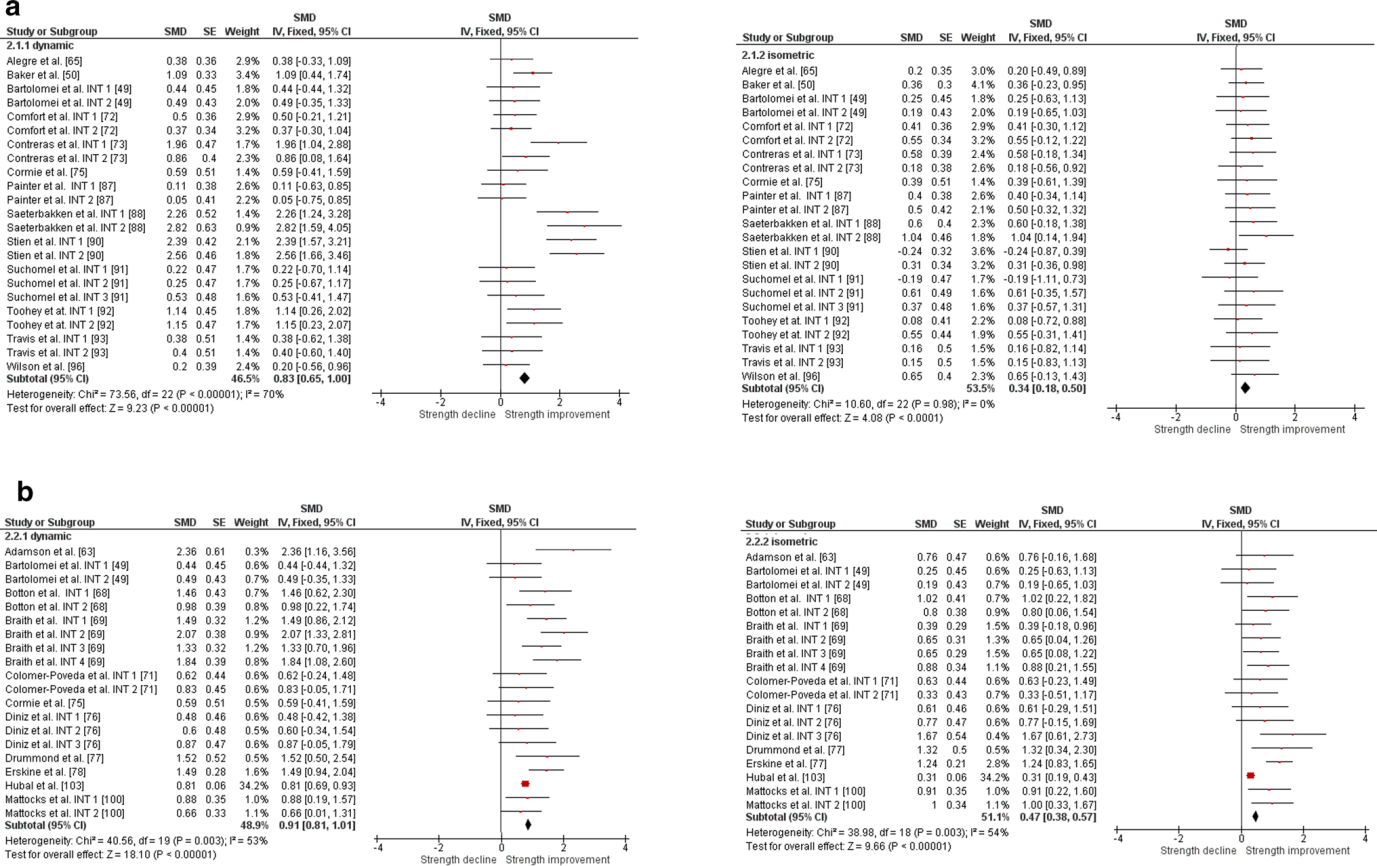
Standardized mean difference (SMD) of conducting dynamic resistance training on task-specificity (dynamic) strength and the transferability of strength (isometric) for **a** position-matched multi-joint exercises and **b** position-matched single-joint exercises. *CI* confidence interval, *df* degrees of freedom, *INT* intervention, *IV* inverse variance, *Random* random-effects model, *SE* standard error

The overall moderate magnitude SMD of dynamic muscle strength following dynamic RT was 0.98 (95% confidence interval 0.91–1.06, *p* < 0.001, *I*^2^ = 67%, *χ*^2^ = 217.73, *df* = 71), whereas the small magnitude SMD of isometric muscle strength amounted to 0.42 (95% confidence interval 0.35–0.49, *p* < 0.001, *I*^2^ = 4%, *χ*^2^ = 74.04, *df* = 71) [Fig. 2]. For the sub-group analyses (e.g., training status, multi-joint or single-joint exercises, intervention duration, upper or lower body exercise, and position matched multi-joint and single-joint exercises in both dynamic and isometric contraction), the dynamic strength displayed moderate-to-large effects (SMD = 0.75–1.30), whereas the isometric strength displayed small-to-moderate effects (SMD = 0.29–0.70, Figs. 3, 4, 5, 6, 7).

### Meta-Regression Analyses

Table 2 shows the results of random-effects meta-regressions for RT-induced hypertrophy and muscle activity changes. None of the mechanistic effects significantly predicted the effects of dynamic RT on dynamic and isometric muscle strength (*p* ≥ 0.222). The explained variance between effect sizes ranged from 0 to 13.4%.

**Table 2:** Results of the random-effects meta-regression analyses for muscle hypertrophy and muscle activity to predict the effects of dynamic resistance training on dynamic and static muscle strength in healthy adults

Covariate	Estimate	SE	95% Confidence interval		*z* value	*p*-Value	*R*^2^	AIC
			Lower	Upper				
*Dynamic muscle strength*								
Model 1.1 (hypertrophy)								
Intercept	1.04	0.24	0.57	1.50	4.37	< 0.001	13.4%	46.6
Hypertrophy effect	0.32	0.34	− 0.34	0.97	0.95	0.342		
Model 1.2 (muscle activity)								
Intercept	1.98	0.26	1.47	2.48	7.66	< 0.001	3.6%	33.9
Muscle activity effect	− 0.35	0.39	− 1.11	0.41	− 0.90	0.367		
*Isometric muscle strength*								
Model 2.1 (hypertrophy)								
Intercept	0.49	0.15	0.21	0.78	3.35	< 0.001	9.5%	26.5
Hypertrophy effect	0.25	0.20	− 0.15	0.65	1.22	0.222		
Model 2.2 (muscle activity)								
Intercept	0.57	0.16	0.26	0.88	3.58	< 0.001	0.0%	22.0
Muscle activity effect	0.21	0.24	− 0.27	0.69	0.86	0.392		

*AIC* Akaike information criterion, *SE* standard error

## Discussion

The primary aim of this systematic review and meta-analysis was to examine the effects of dynamic RT on dynamic (i.e., task specificity) as well as isometric muscle strength (i.e., transferability). In general, the task-specific effects of dynamic RT were moderate (SMD = 0.98, range 0.05–3.06), whereas the transferability effects were small sized (SMD = 0.42, range − 0.24 to 1.67). Furthermore, RT-induced effects on muscle hypertrophy and muscle activity levels did not significantly predict RT-induced effects on dynamic and isometric muscle strength.

The magnitude of the aforementioned findings indicates that changes in muscle strength after conducting a traditional dynamic RT program can drop significantly or be even masked by only using isometric testing. Of note, all included studies used dynamic and isometric testing targeting the same muscle groups. Importantly, of the 72 different RT interventions included, only one study examined a non-trained exercise in the dynamic testing procedures [74]. Therefore, it is possible that the moderate-sized task specificity (i.e., dynamic tests) and the small-sized transferability of dynamic RT are related to (1) the specificity of the contraction form and/or (2) the specificity of the exercise (i.e., movement pattern) being used during testing [5, 12, 109]. Based on the present findings, it is difficult to separate these two explanations because of the heterogeneity of the interventions, subjects, and testing procedures. It has been argued that improvements in lifting technique and muscle coordination may cause a greater strength improvement in multi-joint free-weight exercises than single-joint fixated movements among beginners in RT [5, 12, 74, 91]. However, an improvement in muscle strength has been demonstrated with a different RT status (trained vs sedentary), whereas the complexity of the RT exercises (single-joint vs multi-joint exercises), RT modality (training machines vs free weights), sex, age, and numbers of familiarization sessions can modulate short-term improvements [21, 89, 109, 110]. Recently, several studies have demonstrated good-to-excellent test–retest reliability in dynamic and isometric testing of maximal strength, including different populations (sex, RT experience, age) and exercises (single-joint, multi-joint, upper body, or lower body) [21, 111, 112].

The main findings (task specificity of trained contraction form and its transferability to the non-trained contraction form) are supported by the review by Wilson and Murphy [15] and two more recent meta-analyses by Spitz et al. [3] and James et al. [14]. Still, there are some major differences between the present study and the studies mentioned above. More specifically, James et al. [14] included RT studies that only examined the squat, deadlifts, and power cleans with position-matched isometric muscle strength tests. The authors included 11 studies and demonstrated an overall pooled effect size of 0.13 (trivial effect) in favor of dynamic tests compared to isometric strength. Accordingly, the present study included a similar position-matched sub-group analysis for both multi-joints and single joints as James et al. [14] and demonstrated a moderate SMD for the task-specificity for multi-joint and single-joint exercises (SMD = 0.83 and 0.91) with small transferability to non-trained contraction form (SMD = 0.34 and 0.47). Importantly, and as the most likely explanation of the different findings, James et al. [14] had highly resistance trained as an inclusion criterion while the present study included a population that ranged from beginners to resistance-trained subjects. Furthermore, Spitz et al. [3] included both isokinetic and isometric tests to examine the generality of muscle strength and demonstrated a moderate effect (SMD = 0.80) in the non-trained muscle strength tests in contrast to the present findings of a small effect (SMD = 0.42). Spitz et al. [3] also demonstrated larger task-specificity effects compared with the present study (SMD = 1.84 vs SMD = 0.98). This may be explained by the number of included studies (*n* = 12), the variety in RT interventions included (e.g., blood flow restriction to high-intensity RT), and the heterogeneous populations (i.e., ranging from post-menopausal women aged 54–65 years to trained individual athletes) in Spitz et al. [3]. Notably, Spitz et al. [3] did not include sub-group analyses to identify potential training-related moderators.

Resistance training status demonstrated different task-specificity effects (range SMD; 0.75–1.27) of dynamic RT and only small transferability effects (range SMD; 0.29–0.58). As expected, the resistance trained demonstrated the lowest RT effects of the three groups (training plateau effect) in both dynamic (SMD = 0.75) and isometric (SMD = 0.29) muscle strength tests, whereas untrained individuals demonstrated the largest effects (dynamic; SMD = 1.27, isometric; SMD = 0.58) [ascending portion of the training curve]. These findings are supported by previous findings [113] and can be explained by the “ceiling effect,” which has been proven for strength development [49] and muscle hypertrophy [114]. Still, and irrespective of RT status, the task-specificity effects were more than twice as large as the transferability effects (SMD ratio dynamic: isometric of 2.2–2.6). For the other sub-analyses (i.e., multi-joint testing exercises, intervention duration, body segment), the task specificity demonstrated moderate-to-large effects (range; 1.02–1.24) with only small effects (range; 0.33–0.46) of transferability. This demonstrates that the potential moderators (i.e., multi-joint testing exercises, intervention duration, body segment) did not have a significant impact on the task specificity and transferability. Accordingly, the meta-analysis of James et al. [14] included analyses of the coefficient of variation between changes in position-matched dynamic and isometric strength tests among highly RT subjects. The coefficient of variation was 109%, which demonstrates that there was no predictable relationship between the changes in the two contraction forms at the population level.

The transferability of muscle strength demonstrated a moderate effect (SMD = 0.70) for single-joint exercises and a small effect for the multi-joint exercises (SMD = 0.33), whereas the task specificity was moderate to large (SMD = 1.02 and 1.30) for the multi-joint and single-joint exercises. These findings support the hypotheses presented by Gentil et al. [17]. These authors [17] proposed a hypothesis that the prime movers in a multi-joint exercise do not receive sufficient or optimal stimuli as the synergist muscles might fatigue before the prime movers. This could explain the (1) large effect of transferability of strength for single-joint exercises compared with multi-joint exercises, (2) the larger requirement for technique in multi-joint exercises (e.g., squat), especially for sedentary individuals undertaking RT, or (3) a combination of both factors [91]. It has been suggested that muscle hypertrophy occurs earlier when using single-joint exercises compared with multi-joint exercises because of differences in neuromuscular adaptations between the modalities [5, 115]. The resistive load would be focused on one major muscle group with a single-joint exercise, whereas it would be distributed over a number of muscles with multi-joint RT exercises. Furthermore, Gentil et al. [17] reported no long-term differences in muscle strength or muscle size comparing single-joint and multi-joint RT. Stien et al. [91] conducted an 8-week RT intervention randomizing physically active women into one multi-joint exercise (leg-press) or two single-joint exercises (knee extension and kick-back). It was reported that the greatest 6RM strength gains were observed with the trained exercise(s), but both groups improved the non-trained exercise(s) with no differences in muscle activity between the groups.

Typically, squats, deadlift, or leg press (multi-joint exercises) were used as the dynamic test and knee extensions during the isometric testing conditions. Therefore, comparing a dynamic contraction using a multi-joint exercise with an isometric contraction using a single-joint exercise may potentially be influenced by the (1) contraction form and (2) the number of joints involved in the task. Therefore, we conducted additional analyses comparing multi-joint versus multi-joint (25 interventions), multi-joint versus single-joint (28 interventions), and single versus single-joint (19 interventions) exercises. For the trained dynamic contraction (i.e., task specificity), these analyses demonstrated moderate effects (SMD = 0.76–1.19) for multi-joint versus multi-joint, multi-joint versus single-joint, and single-joint versus single-joint exercises, but only small effects (SMD = 0.32–0.49) for the transferability to the isometric non-trained contraction form. Greater complexity of multi-joint exercises (number of muscle groups involved, stability requirement, and muscle synchronizations) than single-joint exercises may explain the lower effect of the multi-joint versus multi-joint comparison compared with the large effect of multi-joint versus single or single versus single-joint exercises. The lower effects of comparing multi-joint versus single joint were supported by James et al. [14] who examined the effects of changes in squat, deadlift, and power clean and position-matched isometric strength (mid-thigh pull and isometric squat) and demonstrated an overall pooled effect (SMD) of 0.13 in favor of dynamic testing. However, James et al. [14] included highly resistance trained subjects (CrossFit, powerlifters, athletes), which may explain the lower task specificity than the present findings.

Both morphological (i.e., hypertrophy) and neural adaptations following RT interventions have been examined [5, 116–118]. The debate regarding contributions to muscle strength gains for individuals with a different RT status and the short-term and long-term adaptations is continuing [5, 116]. However, it could be speculated that morphological and neuromuscular adaptations (i.e., muscle hypertrophy and muscle activity changes, respectively) may affect the dynamic and isometric muscle strength differently. For example, isometric strength has been referred to as an individual’s “pure strength” capacity owing to the limited requirements of intra-muscular and inter-muscular coordination [30]. In contrast, dynamic multi-joint exercises require a substantial intra-muscular and inter-muscular coordination, which has been illustrated by short-term strength improvements exceeding concomitant muscle hypertrophy [5, 89, 91, 109]. The present study’s findings did not support these speculations as RT-induced hypertrophy and muscle activity changes predicted the effects of dynamic RT on dynamic and isometric muscle strength. Of note, only a limited number of the included studies reported morphological (*n* = 13) and neural (*n* = 9) adaptations. The heterogeneity between these studies in terms of population (RT status, age, and sample size), training intervention (length, volume, and intensity), and methods (magnetic resonance imaging, ultrasound, electro-stimulation, normalization of muscle activity) need to be taken into account during interpretation. In particular, the methods examining pre-test to post-test changes in muscle activity with RT interventions have been debated [119]. Importantly, the individuals included in the present meta-analysis were the same participants conducting both testing conditions. Therefore, differences in muscle hypertrophy could not necessarily explain the differences in task specificity and transferability of dynamic RT as the muscle hypertrophy will affect both testing conditions. Hence, greater task-specificity effects suggest that the neural adaptations may be the cause, as supported by previous findings demonstrating the significance of repeat training and testing the same exercise [2, 5, 109, 110]. The predominance of neural adaptations for the increases in strength during the first 8–12 weeks of a RT program has been suggested for over 40 years [8, 12, 117, 120]. As the majority of studies in this review were of 12 weeks duration or less, the relative contributions of muscle hypertrophy may have been diminished when compared with longer duration training programs. More recently, the acute changes in muscle shape (i.e., pennation angle and fascicle length) were examined in fixed-end contractions (isometric at the muscle–tendon unit level) and dynamic contractions in recreationally active runners [42]. Resting fascicle angles was more strongly correlated with the rate of force development during the dynamic contraction than the isometric contraction because of different geometric restrictions in the muscle shape and architectural gearing. Importantly, the study was cross-sectional and cannot be generalized to longitudinal RT adaptations, a longer duration of the contractions, or other populations. Still, the findings by van Hooren et al. [42] may propose a possible explanation for the differences observed between dynamic and isometric actions demonstrated in the present findings.

Including isometric strength testing in dynamic RT interventions may have several significant and practical applications. For example, it has been argued that isometric testing is more safe than dynamic testing  [30] and causes less fatigue than dynamic testing [121]. Furthermore, adaptations such as the rate of force development and the impulse of specific joint angles are impossible to detect without isometric tests, in addition to the possibility of quantifying force–time characteristics [15, 38]. A recent systematic review demonstrated a small-to-large correlation between dynamic and isometric bench press performance (*r* = 0.118–0.700) [38], which corresponds to a shared variance of *r*^2^ = 1–49. It therefore seems reasonable to assume that these forms of muscle strength represent different domains or capacities or the selected joint angle during the isometric testing. Lum et al. [19] suggested that the joint angle at which the isometric test should be performed is the joint angle where the greatest dynamic force is developed. In the present study, a trivial shared variance was demonstrated between task specificity (dynamic contraction) and transferability of dynamic RT using either the effect sizes (*r*^2^ = 0.08) or the percentage of pre-test to post-test changes (*r*^2^ = 0.14). These findings were supported by James et al. [14]. These results demonstrate a considerable weaker association between the two testing conditions than reported between strength and power testing in cross-sectional studies [122, 123]. Therefore, including isometric tests in a traditional RT intervention may represent an output of maximal strength, but represents different qualities than the dynamic muscle strength testing. The weak longitudinal association between dynamic and isometric tests could be due to different underpinning mechanisms. For example, the stretch–shortening cycle is normally present only during dynamic contractions [124], whereas it has been suggested that musculoskeletal stiffness may contribute significantly to the force output during isometric tasks [125]. The associations between these mechanisms (i.e., stretch–shortening cycle and muscle stiffness) and changes in dynamic and isometric muscle strength need to be examined further to fully understand the neuromuscular adaptation to the different contraction forms.

There are some limitations in the present paper that need to be acknowledged. Because of the nature of our research question, locating papers was challenging. None of the included papers was designed with the primary aim of comparing the changes in dynamic and isometric strength. Therefore, we had to widen our search terms, which resulted in 10,312 papers being screened. It is possible that potentially relevant papers were excluded during this comprehensive screening process. However, we identified and included more papers than previous comparable meta-analyses [3, 14] even though our research question deviated from the previous one. Importantly, including studies with different RT statuses, interventions, modalities, and upper and lower body focus resulted in great heterogeneity among the studies. However, conducting sub-group analyses of these potential moderators represents a novel feature of the present study. Study quality assessment scores indicated a good methodological quality. Of note, the most common criterion not to be met was the inclusion of a control group. The present meta-analysis did not include between-group comparisons (i.e., training vs control group), but did evaluate within-group improvements in the two testing conditions. Still, we cannot ignore the possibility that different methodological approaches contributed to different effects of task specificity and the transferability of dynamic RT. This may explain the observed evidence of considerable heterogeneity displayed in the present analysis, which ranged from low to high (*I*^2^ = 0–79%) with > 75% rated as considerable [62].

## Conclusions

The main analysis demonstrated a moderate-sized task-specificity effect of dynamic RT on dynamic muscle strength compared with only small transferability effects on isometric muscle strength. In general, similar findings to those of the main analysis were demonstrated in the sub-group analysis. Of the different moderators, sedentary subjects and single-joint exercises had the largest impact on task specificity. The limited association between task specificity and transferability of dynamic RT suggested that dynamic and isometric muscle strength represent different strength domains. Muscle hypertrophy and changes in muscle activity following dynamic RT could not predict RT-induced dynamic and isometric strength gains in healthy adults.

## Declarations

### Availability of Data and Material

The datasets generated and/or analyzed during the current study will be publicly available in Open Science Framework.
